# In Memoriam—Thos. F. Pomeroy, M. D.

**Published:** 1892-05

**Authors:** 


### IN MEMORIAM.

### Thomas F. Pomeroy, M. D.

Once more The Homœopathic Physician is called upon to chronicle the death of one of the older practitioners of pure Homœopathy—Dr. Thomas F. Pomeroy. He departed this life April 2d, 1892, at his residence, 758 High Street, Providence, Rhode Island.

He was born May 11th, 1816, in Cooperstown, Otsego County, New York. His father, the late Theodore Pomeroy, M. D., of Utica, and his maternal grandfather, the late Thomas Fuller, M. D., of Cooperstown, practiced medicine for many years, and won for themselves a high reputation in their profession. After several years spent in preparatory and collegiate education, Dr. Pomeroy graduated at Union College, Schenectady, in 1836. Commencing the study of medicine, he attended two full courses of lectures at the Cleveland homeopathic medical college, and graduated in the spring of 1853. In May he began practice in Utica, in company with Lucian B. Wells, M. D., an old friend, and a former student of his father. In 1859

he removed to Detroit, where he successfully filled the duties of his profession until impaired health made a change necessary. He was a firm believer in the law of cure as promulgated by Hahnemann, and his practice was in accordance therewith. He was a member of the International Hahnemannian Association, and an honorary senior member of the American Institute of Homœopathy. He was the First Vice-President of the Homœopathic Medical Society of the State of Michigan, for one year, and its President the year following, and an occasional contributor to some of the medical journals. For several years past he had been a great sufferer from chronic diseases, which he endured with patience. Shortly before his death, he became unconscious, and on the second day of April a life in which he had his full share of pain was ended.

Dr. Pomeroy was for many years the leading homœopathic physician of Detroit. His failing health compelled the total relinquishment of his practice, and so he moved about from one city to another, never staying long in any one place.

He was a warm friend of the late Dr. Adolph Lippe, and when not visiting in Philadelphia he was in constant correspondence with Dr. Lippe, who was also his medical attendant.

In the spring of 1891 he made a visit to Philadelphia, and summoned the editor of this journal as his medical adviser in a ferocious attack of renal colic. Lycopodium was the remedy. But the patient at first refused to take it, declaring it was not indicated. He finally consented, and the remedy gave relief in twenty minutes. Then came an attack, apparently, of angina pectoris, for which Lachesis was prescribed. This, too, he took under protest, but it also gave relief. After this experience he facetiously dubbed his doctor "LL. D." (Lachesis Lycopodium Doctor).

In the summer, being now quite restored to his usual state of health, which, as before stated, was not good, he went to Providence. There repeated attacks of first one complaint and then another prostrated him completely, and thus brought his life to an end, within six weeks of being seventy-six years old.

His physician in Providence was Dr. Henry A. Whitmarsh, of whose attentions to him in his last illness his family speak with gratitude.

Although for many years not a practitioner, he took a strong interest in everything connected with medicine, and kept up a lively correspondence with Dr. Lee and the present editor, and several other friends. He was also much interested in politics, being a Democrat, and his letters were noted for the intensity of his convictions on both medicine and politics, and the boldness of his utterances in regard to them.

He leaves a wife, son, and three daughters. W. M. J.

